# Natural Antioxidants: Determination in Food and Nutraceuticals and Implications on Human Health

**DOI:** 10.3390/biotech15010010

**Published:** 2026-01-21

**Authors:** Gregorio Peron

**Affiliations:** Department of Molecular and Translational Medicine, University of Brescia, 25123 Brescia, Italy; gregorio.peron@unibs.it

Oxidative processes influence several aspects of biology, from the subtle balance of redox signaling to the destructive cascade of oxidative damage associated with chronic disease and aging. In this context, natural antioxidants, i.e., bioactive compounds derived from food matrices and nutraceutical sources, have attracted scientific and public interest for their potential in promoting health, preserving food quality, and mitigating adverse outcomes associated with oxidative stress [1]. This Special Issue (SI), titled “Natural Antioxidants: Determination in Food and Nutraceuticals and Implications for Human Health”, was conceived to address both foundational and translational aspects of this rapidly evolving field. The significance of natural antioxidants lies in their multifaceted roles. Chemically, they are capable of neutralizing reactive molecular species, thereby reducing direct oxidative damage to lipids, proteins, and nucleic acids. Biologically, they influence endogenous defense systems through modulation of redox-sensitive signaling pathways and detoxifying enzymes [2]. Furthermore, the metabolic interplay between dietary antioxidants and the gut microbiota suggests complex systemic effects that extend beyond direct free-radical scavenging [3]. However, despite widespread interest, their mechanisms of action and physiological relevance remain areas of active investigation and debate.

A fundamental aspect of antioxidant research relies on the accurate determination and characterization of these compounds in food and nutraceuticals. Reliable analytical strategies (ranging from chromatographic separation to advanced spectrometric profiling) are required for defining antioxidant composition, quantifying bioactive components, and assessing their stability during food processing, storage, and digestion. This SI has collected contributions that reinforce the critical link between methodological rigor and meaningful biological interpretation. Across diverse food matrices and biological models, the findings presented in the SI highlight the diversity of antioxidant phenotypes, but also the necessity of integrative analytical frameworks to reveal their functional potential.

From a technological perspective, insights into how processing and preparation influence antioxidant profiles were emphasized. Natural products often undergo modifications during plant germination [4], extraction procedures, or enzymatic treatment [5], which can enhance or impair antioxidant capacity. Understanding the effects of such manipulations is crucial, both for the optimization of functional food ingredients and for preserving the intrinsic nutritional value of traditional dietary sources. These considerations extend to the formulation of nutraceuticals where extraction, fractionation, and delivery systems determine their bioavailability and biological efficacy in vivo.

Interdisciplinary approaches for studying antioxidants are presented in this SI, ranging from phytochemical quantification to microbial metabolite profiling and in vivo bioactivity assessments. The collective work clearly demonstrates that effective antioxidant research should integrate diverse yet complementary disciplines such as analytical chemistry, food science, microbiology, and biological evaluation. This integrative perspective is appropriate because antioxidant function cannot be fully understood solely through chemical assays. Indeed, it requires contextualization within complex biological systems, including cellular models, animal models, and whole-organism responses.

The human health implications of natural antioxidants remain a central focus. Oxidative stress is involved in the pathogenesis of cardiovascular diseases, metabolic disorders, neurodegeneration, and cancer [2]. Natural antioxidants, whether delivered through diet or nutraceutical supplementation, are often promoted for their purported preventive or therapeutic benefits. However, elucidating how these compounds behave physiologically remains an ongoing challenge. The contributions in this SI explore aspects of this continuum, including the dynamics of antioxidant uptake and clearance, and modulation of enzymatic defenses in living organisms, thus providing valuable data to inform hypotheses on their health relevance.

Another aspect of antioxidant research covered in this SI is the relationship between natural antioxidants and the gut microbiome. The transformation of dietary antioxidants by intestinal microbes can yield metabolites with distinct bioactivities, suggesting that the health implications of antioxidant intake cannot be fully appreciated without considering the role of microbial metabolism. This emerging area bridges nutritional biochemistry and microbiome science, pointing toward more nuanced conceptual models of how dietary components influence systemic physiology.

In addition to their health benefits, natural antioxidants play important roles in food quality and preservation. Oxidative degradation can compromise sensory attributes, nutritional value, and shelf life. Antioxidants can contribute to food stability and consumer appeal by mitigating lipid peroxidation and preserving functional compounds. This dual relevance to food science and health highlights the interconnected nature of research in this domain.

Taken together, the contributions to this SI reflect a comprehensive exploration of antioxidants from chemical determination to biological implication. They demonstrate that progress in this field depends on methodological excellence in analytical determination, conceptual clarity about biological mechanisms, and translational insight into human health implications. By emphasizing diverse food sources and analytical strategies, and integrative biological approaches, this collection advances our understanding of how natural antioxidants can be characterized, optimized, and potentially leveraged for health promotion. As the scientific community continues to refine analytical tools and deepen biological models, future research will further clarify the conditions under which natural antioxidants exert health effects in vivo, how they interact with complex biological systems, and how food science innovations can enhance their functional potential.

Finally, as the Guest Editor, I would like to thank all the authors who contributed to this SI by publishing their research, as well as the reviewers and the Assistant and Academic Editors for their valuable support.
